# Hadronic uncertainties versus new physics for the *W* boson mass and Muon *g* − 2 anomalies

**DOI:** 10.1038/s41467-023-36366-7

**Published:** 2023-02-07

**Authors:** Peter Athron, Andrew Fowlie, Chih-Ting Lu, Lei Wu, Yongcheng Wu, Bin Zhu

**Affiliations:** 1 Department of Physics and Institute of Theoretical Physics, Nanjing Normal University, Nanjing, 210023 China; 2 Department of Physics, Yantai University, Yantai, 264005 China

**Keywords:** Phenomenology, Theoretical particle physics

## Abstract

There are now two single measurements of precision observables that have major anomalies in the Standard Model: the recent CDF measurement of the *W* mass shows a 7*σ* deviation and the Muon *g* − 2 experiment at FNAL confirmed a long-standing anomaly, implying a 4.2*σ* deviation. Doubts regarding new physics interpretations of these anomalies could stem from uncertainties in the common hadronic contributions. We demonstrate that these two anomalies pull the hadronic contributions in opposite directions by performing electroweak fits in which the hadronic contribution was allowed to float. The fits show that including the *g* − 2 measurement worsens the tension with the CDF measurement and conversely that adjustments that alleviate the CDF tension worsen the *g* − 2 tension beyond 5*σ*. This means that if we adopt the CDF *W* mass measurement, the case for new physics in either the *W* mass or muon *g* − 2 is inescapable regardless of the size of the SM hadronic contributions. Lastly, we demonstrate that a mixed scalar leptoquark extension of the Standard Model could explain both anomalies simultaneously.

## Abstract (web summary)

The tension between measured W mass and its Standard Model prediction might arise from uncertainties in the hadronic contribution, and the same is true for the muon g − 2. Here, the authors show that such a common origin for the two anomalies is unlikely, while a model involving leptoquarks might explain them both.

## Introduction

The CDF collaboration at Fermilab recently reported the world’s most precise direct measurement of the *W* boson mass, ${M}_{W}^{{{{{{{{\rm{CDF}}}}}}}}}=80.4335\pm 0.0094\,{{{{{{{\rm{GeV}}}}}}}}$^1^, based on 8.8/fb of data collected between 2002–2011. This deviates from the Standard Model (SM) prediction by about 7*σ*. The recent FNAL E989 measurement of the muon’s anomalous magnetic moment furthermore implies a new world average of *a*_*μ*_ = 16 592 061(41) × 10^−11^ ^2^, which is in 4.2*σ* tension with the SM theory prediction from the Muon *g* − 2 Theory Initiative, 116 591 810(43) × 10^−11^ ^3^. This prediction is based on results from refs. ^4–29^.

**Fig. 1:**
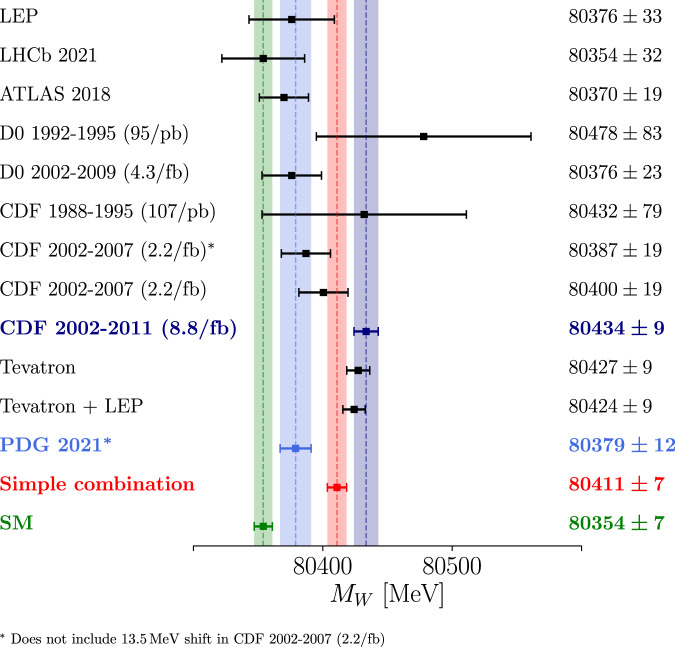
Simple combination of *M*_*W*_ measured by different experiments. Measurements of the *W* boson mass from LEP^105^, LHCb^106^, ATLAS^107^, D0^108^ and CDF I and II^1,31,109^. We show an SM prediction^66^, the previous PDG combination of measurements^110^, CDF combinations of Tevatron and LEP measurements^1^, and a simple combination that includes the new measurement, which is explained in the Supplementary Note 1. The PDG combination includes uncorrected CDF measurements. The error bars show 1*σ* errors. The code to reproduce this figure is available at ref. ^111^.

Whilst the Fermilab *g* − 2 measurement was in agreement with the previous BNL E821 measurement^30^, as shown in Fig. 1 there appears to be tension between the new CDF measurement and previous measurements, including the previous CDF measurement with only 2.2/fb of data^31^. Updates to systematic uncertainties shift the previous measurement by 13.5 MeV, however, such that the CDF measurements are self-consistent. In the Supplementary Note 1 we find a reduced chi-squared from a combination of *N* = 7 measurements of about *χ*^2^/(*N* − 1) ≃ 3 and a tension of about 2.5*σ*. Nevertheless, we show that these two measurements could point towards physics beyond the SM with a common origin and, under reasonable assumptions, that the new CDF *W* mass measurement pulls common hadronic contributions in a direction that significantly strengthens the case for new physics in muon *g* − 2.

We now turn to the SM predictions for the *W* mass and muon *g* − 2. Muon decay can be used to predict *M*_*W*_ in the SM from the more precisely measured inputs, *G*_*μ*_, *M*_*Z*_ and *α* (see e.g. ref. ^32^)

1 $${M}_{W}^{2}={M}_{Z}^{2}\left\{\frac{1}{2}+\sqrt{\frac{1}{4}-\frac{\pi \alpha }{\sqrt{2}{G}_{\mu }{M}_{Z}^{2}}\left(1+\Delta r\right)}\right\}.$$

The loop corrections are contained in Δ*r*: full one-loop contributions were first calculated in refs. ^33,34^, and the complete two-loop contributions are now available^35–52^. These have been augmented with leading three-loop and leading four-loop corrections^53–62^. The state-of-the-art on-shell (OS) calculation of *M*_*W*_ in the SM^32^ updated with recent data gives 80.356 GeV^63^, whereas the $\overline{{{{{{{{\rm{MS}}}}}}}}}$ scheme^64^ result is about 6 MeV smaller when evaluated with the same input data. Direct estimates of the missing higher order corrections were a little smaller (4 MeV for OS and 3 MeV for $\overline{{{{{{{{\rm{MS}}}}}}}}}$).

The predictions also suffer from parametric uncertainties, with the largest uncertainties coming from *m*_*t*_ and may be around 9 MeV^64^, and depend on estimates of the hadronic contributions to the running of the fine structure constant, $\Delta {\alpha }_{{{{{{{{\rm{had}}}}}}}}}\equiv \Delta {\alpha }_{{{{{{{{\rm{had}}}}}}}}}^{(5)}({M}_{Z}^{2})$, defined at the scale *M*_*Z*_ for five quark flavours. This is constrained by electroweak (EW) data and by measurements of the *e*^+^*e*^−^ → *hadrons* cross section (*σ*_had_) through the principal value of the integral^65^

2 $$\Delta {\alpha }_{{{{{{{{\rm{had}}}}}}}}}=\frac{{M}_{Z}^{2}}{4{\pi }^{2}\alpha }\,{-}\!\!\!\!\!\!\int\nolimits_{{m}_{{\pi }^{0}}^{2}}^{\infty }\frac{{{{{{{{\rm{d}}}}}}}}s}{{M}_{Z}^{2}-s}\,{\sigma }_{{{{{{{{\rm{had}}}}}}}}}(\sqrt{s}),$$

where ${m}_{{\pi }^{0}}$ is the neutral pion mass. The parametric uncertainties may be estimated through global EW fits. For example, two recent global fits without any direct measurements of the *W* boson mass predict 80.354 ± 0.007 GeV^66^ and 80.3591 ± 0.0052 GeV^67^ in the OS scheme. Lastly, the CDF collaboration quote 80.357 ± 0.006 GeV^1^. While the precise central values and uncertainty estimates vary a little, all of these predictions differ from the new CDF measurement by about 7*σ*.

Turning to muon *g* − 2, the SM prediction for *a*_*μ*_includes hadronic vacuum polarization (HVP) and hadronic light-by-light (HLbL) contributions in addition to the QED and EW contributions that can be calculated perturbatively from first principles^3^. Although HVP is not the main contribution for *a*_*μ*_, it suffers from the largest uncertainty and it is hard to pin down its size. The HLbL contributions in contrast have a significantly smaller uncertainty, with data-driven methods now providing the most precise estimates but with lattice QCD results that are consistent with these and which also contribute to the final result in ref. ^3^. Two approaches are commonly used to extract the contributions from HVP. First, a traditional data-driven method in which the HVP contributions are determined from measurements of *σ*_had_ using the relationship^68^

3 $${a}_{\mu }^{{{{{{{{\rm{HVP}}}}}}}}}=\frac{{m}_{\mu }^{2}}{12{\pi }^{3}}\int\nolimits_{{m}_{{\pi }^{0}}^{2}}^{\infty }\frac{{{{{{{{\rm{d}}}}}}}}s}{s}K(s)\,{\sigma }_{{{{{{{{\rm{had}}}}}}}}}(\sqrt{s}),$$

where *m*_*μ*_ and ${m}_{{\pi }^{0}}$ are the muon and neutral pion masses, respectively, and *K*(*s*) is the kernel function as shown in refs. ^68,69^. This approach results in ${a}_{\mu }^{{{{{{{{\rm{HVP}}}}}}}}}=693.1(4.0)\times 1{0}^{-10}$ with an uncertainty of <0.6%^8–10,12,13,70^. The second approach uses lattice QCD calculations. The recent leading-order lattice QCD calculations for HVP from the BMW collaboration significantly reduced the uncertainties and resulted in ${a}_{\mu }^{{{{{{{{\rm{HVP}}}}}}}}}=707.7(5.5)\times 1{0}^{-10}$ ^71^. This, however, shows tension with the *σ*_had_ measurements method.

The *M*_*W*_ and muon *g* − 2 calculations are in fact connected by the fact that both Δ*α*_had_ and the HVP contributions can be extracted from the hadronic cross section, ${\sigma }_{{{{{{{{\rm{had}}}}}}}}}(\sqrt{s})$, through eqs. (2) and (3). We assume that the energy dependence of this cross-section, $g(\sqrt{s})$, is reliably known for $\sqrt{s}\ge {m}_{{\pi }^{0}}$^9,12^, but that the overall scale, *σ*_had_, may be adjusted,

4 $${\sigma }_{{{{{{{{\rm{had}}}}}}}}}(\sqrt{s})={\sigma }_{{{{{{{{\rm{had}}}}}}}}}\,g(\sqrt{s}).$$

This simple modification is similar to scenario (3) in ref. ^65^. There are of course more complicated possibilities, including increases and decreases in the hadronic cross section at different energies. Reference ^72^ considered these complicated possibilities to be implausible, though this is a somewhat subjective matter; see Supplementary Note 2 for further discussion. Using eqs. (2) and (4) we may trade *σ*_had_ for Δ*α*_had_ giving Δ*α*_had_ ∝ *σ*_had_. The HVP contributions depend on Δ*α*_had_ and conversely estimates of the HVP contributions from either hadronic cross-sections or lattice QCD constrain Δ*α*_had_. Further details of the transformation between Δ*α*_had_ and ${a}_{\mu }^{{{{{{{{\rm{HVP}}}}}}}}}$are provided in the Supplementary Note 2. Thus we can transfer constraints on Δ*α*_had_ from measurements of *M*_*W*_ to constraints on the HVP contributions to muon *g* − 2 and vice-versa^72–74^ through global EW fits.

In this work, we study how the new *M*_*W*_ measurement from CDF impacts estimates of muon *g* − 2 in global EW fits and show that a common explanation of muon *g* − 2 and the CDF *M*_*W*_ from hadronic uncertainties are not possible. Then we demonstrate that in contrast a scalar leptoquark model could provide a simultaneous explanation of both muon *g* − 2 and the *W* mass anomalies.

## Results and discussion

### Electroweak Fits of the *W* mass and Muon *g* − 2

**Fig. 2:**
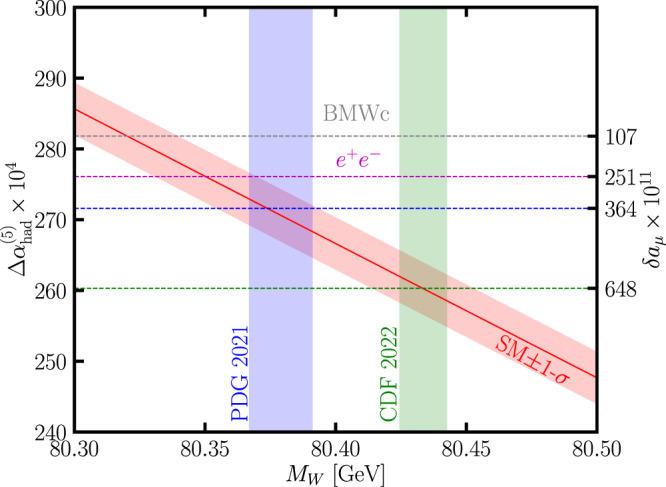
Correlation of Δ*α*_had_ with *M*_*W*_ in the electroweak fits. The allowed values of Δ*α*_had_ assuming specific values of the *W* boson mass in the SM found from EW fits. The solid line indicates the central value from the fit without any input for Δ*α*_had_, while the red band shows the 1*σ* region. The current world average (PDG 2021) and new measurement (CDF 2022) for *M*_*W*_ are indicated by vertical bands. We indicate the favoured Δ*α*_had_ from BMW lattice calculations (grey), *e*^+^*e*^−^ cross section measurements (magenta), our fit using *M*_*W*_ from PDG 2021 (blue) and our fit using *M*_*W*_ from CDF 2022 (green).

We first investigated the impact of the *W* mass on the allowed values of Δ*α*_had_ by performing EW fits using Gfitter^66,75–78^ with data shown in Supplimentary Table 1 where *m*_*h*_, *m*_*t*_, *M*_*Z*_, *α*_*s*_ and Δ*α*_had_ were allowed to float. The Fermi constant *G*_*F*_ = 1.1663787 × 10^−5^ GeV^−2^ and the fine-structure constant *α* = 1/137.035999074^79^ in the Thompson limit were fixed in our calculation. Although Δ*α*_had_ is not a free parameter of the SM as it is in principle calculable, it isn’t precisely known and so we allowed it to float, following the approach used in ref. ^65^. We found the allowed Δ*α*_had_ when assuming specific *W* masses between 80.3 GeV and 80.5 GeV; the results form the diagonal red band in Fig. 2. We fixed Δ*M*_*W*_ = 9.4 MeV when obtaining the ± 1*σ* region. The previous world average (PDG 2021) and current CDF measurement (CDF 2022) of the *W* mass are shown by blue and green vertical bands, respectively, and the corresponding best-fit Δ*α*_had_ are indicated by blue and green dashed horizontal lines, respectively. From the intersection of regions allowed by CDF 2022 (green) and the EW fit (red), we see that the CDF measurement pulls Δ*α*_had_ down to about 260 × 10^−4^, making the muon *g* − 2 discrepancy even worse. Indeed, unless the CDF measurement is entirely disregarded it must increase the tension between the muon *g* − 2 measurements and the SM prediction. The overall best-fits were found at around *M*_*W*_ ≃ 80.35 GeV, in agreement with previously published fits.

We further scrutinize the impact of assumptions about the HVP contributions and the *W* mass through several fits shown in Table 1. In the first three fits, the *W* mass is only indirectly constrained by EW data, and Δ*α*_had_ is constrained by the BMWc determination of the HVP contributions, by the *e*^+^*e*^−^ data, and indirectly by EW data. The second and final three fits are similar, though the *W* mass is constrained by the PDG 2021 world average and by the CDF 2022 measurement, respectively. In each case we show the overall goodness of fit, and how much the best-fit muon *g* − 2 and *W* mass predictions deviate from the world average and the recent CDF measurement, respectively. Regardless of the constraints imposed on Δ*α*_had_, including the CDF measurement results in poor overall goodness of fit and increases the tension between the SM prediction for *g* − 2 and the world average. The tension between the SM prediction for the *W* mass and the CDF measurement range from 3.2*σ* to 7.8*σ*. However, the former occurs only when estimates of HVP are completely ignored (final column) and at the expense of increased tension in the SM *g* − 2 prediction and a poor overall goodness of fit. Note that this includes the scenario where we do not include any input values for *M*_*W*_ or Δ*α*_had_ in the global EW fit, as shown in the third data column (of twelve). Even in this case there is still a large tension with the CDF measurement (5.8*σ*), indicating that other EW observables also constrain Δ*α*_had_. Using the *e*^+^*e*^−^ estimates of HVP, which is a standard choice, we see about 5*σ* tension in both *g* − 2 and the *W* mass. In fact, the CDF measurement takes the tension between the SM prediction for muon *g* − 2 and the measurements slightly beyond 5*σ*. Switching to BMWc estimates of HVP partially alleviates the tension in *g* − 2 but results in increased tension with the CDF *W* mass measurement.

**Table 1:** SM predictions from EW fits for Δ*α*_had_ and *M*_*W*_, and the differences with respect to measurements of muon *g* − 2 and the *W* mass, *δa*_*μ*_ and $\delta {M}_{W}\equiv {M}_{W}^{{{{{{{{\rm{CDF}}}}}}}}}-{M}_{W}$

*M*_*W*_		Indirect			PDG 2021			CDF 2022			Simple Combination		
Δ*α*_had_		BMWc	*e*^+^*e*^−^	Indirect	BMWc	*e*^+^*e*^−^	Indirect	BMWc	*e*^+^*e*^−^	Indirect	BMWc	*e*^+^*e*^−^	Indirect
Input	*M*_*W*_ [GeV]	–	–	–	80.379(12)	80.379(12)	80.379(12)	80.4335(94)	80.4335(94)	80.4335(94)	80.411(7)	80.411(7)	80.411(7)
	Δ*α*_had_ × 10^4^	281.8(1.5)	276.1(1.1)	–	281.8(1.5)	276.1(1.1)	–	281.8(1.5)	276.1(1.1)	–	281.8(1.5)	276.1(1.1)	–
	*χ*^2^/d*of*	18.32/15	16.01/15	15.89/14	23.41/16	18.74/16	17.59/15	74.51/16	62.58/16	47.19/15	62.08/16	49.79/16	35.48/15
	*M*_*W*_ [GeV]	80.348(6)	80.357(6)	80.359(9)	80.355(6)	80.361(6)	80.367(7)	80.375(5)	80.380(5)	80.396(7)	80.377(5)	80.381(5)	80.393(6)
	Δ*α*_had_ × 10^4^	280.9(1.4)	275.9(1.1)	274.4(4.4)	280.3(1.4)	275.6(1.1)	271.7(3.8)	278.6(1.4)	274.7(1.0)	260.9(3.6)	278.5(1.4)	274.6(1.0)	262.3(3.4)
Fitted	*δa*_*μ*_ × 10^11^	–	–	294(166)	146(68)	264(59)	364(145)	188(68)	289(57)	648(137)	191(68)	289(57)	597(132)
	Tension	–	–	1.8*σ*	2.1*σ*	4.5*σ*	2.5*σ*	2.8*σ*	5.1*σ*	4.7*σ*	2.8*σ*	5.1*σ*	4.5*σ*
	*δM*_*W*_ [MeV]	86(11)	77(11)	75(13)	79(11)	73(11)	67(12)	59(11)	54(11)	38(12)	57(11)	53(11)	41(11)
	Tension	7.8*σ*	7.0*σ*	5.8*σ*	7.2*σ*	6.6*σ*	5.6*σ*	5.4*σ*	4.9*σ*	3.2*σ*	5.2*σ*	4.8*σ*	3.7*σ*

The input data for Δ*α*_had_ and *M*_*W*_ are listed in first two rows for each case.

In summary, our fits showed the extent to which the new *W* mass measurement worsens tension with muon *g* − 2, using the reasonable assumption that the energy-dependence of the hadronic cross section that connects these is well-known and not modified by for example very light new physics. The anomalies pull Δ*α*_had_ in opposite directions in EW fits, making it even harder to explain both within the SM. We thus now turn to a new physics explanation.

### Interpretation in Scalar Leptoquark model

Even without light new physics, sizable BSM contributions to muon *g* − 2 can be obtained by an operator that gives an internal chirality flip in the one-loop muon *g* − 2 corrections (see e.g. refs. ^80,81^ for a review). On the other hand, BSM contributions to the *W* mass can be obtained when there are large corrections to the oblique parameter *T*^82^. We show that a scalar leptoquark model can satisfy both of these criteria and provide a simultaneous explanation of both muon *g* − 2 and the *W* mass anomalies. We anticipate other possibilities, including composite models with non-standard Higgs bosons^83^.

Scalar leptoquarks (LQs) (see ref. ^84^ for a review), or more specifically the scalar leptoquarks referred to as *S*_1_$(\overline{3},1,1/3)$ and *R*_2_(3, 2, 7/6) in refs. ^85–87^, are well known to provide the chirality flip needed to give a large contribution to *a*_*μ*_^88^, and have also been proposed for a simultaneous explanation of the flavour anomalies^89^. Furthermore due to the mass splitting between its physical states the SU(2) doublet *R*_2_ is capable of making a considerable contribution to the *W* mass. However we find that the mass splitting from a conventional Higgs portal interaction cannot generate corrections big enough to reach the CDF measurement, unless the interaction ${\lambda }_{{R}_{2}H}{R}_{2}^{{{{\dagger}}} }H{H}^{{{{\dagger}}} }{R}_{2}$ is non-perturbative. We thus analyze the plausibility of situations in which one-loop contributions to the anomalous muon magnetic moment and *W* mass corrections are created via the mixing of two scalar LQs through the Higgs portal. For simplicity, we consider the *S*_1_*&S*_3_$(\overline{3},3,1/3)$ scenario,

5 $${{{{{{{{\mathcal{L}}}}}}}}}_{{S}_{1}\&{S}_{3}}={{{{{{{{\mathcal{L}}}}}}}}}_{{{{{{{{\rm{mix}}}}}}}}}+{{{{{{{{\mathcal{L}}}}}}}}}_{{{{{{{{\rm{LQ}}}}}}}}},$$

where the first term is responsible for the mixing of the two LQs, and the second specifies the interaction between quarks and leptons

6 $${{{{{{{{\mathcal{L}}}}}}}}}_{{{{{{{{\rm{mix}}}}}}}}}=\lambda {H}^{{{{\dagger}}} }\left(\overrightarrow{\tau }\cdot \vec{{S}_{3}}\right)H{S}_{1}^{*}+{{{{{{{\rm{h.c.}}}}}}}}$$

7 $${{{{{{{{\mathcal{L}}}}}}}}}_{{{{{{{{\rm{LQ}}}}}}}}}={y}_{R}^{ij}{\bar{u}}_{Ri}^{C}{e}_{Rj}{S}_{1}+{y}_{L}^{ij}{\bar{Q}}_{i}^{C}i{\tau }_{2}\left(\overrightarrow{\tau }\cdot {\overrightarrow{S}}_{3}\right){L}_{j}+{{{{{{{\rm{h.c.}}}}}}}}$$

Although a coupling between *S*_1_ and the left-handed lepton and quark fields is also allowed, we do not initially consider it here. Instead we show that it is possible to have new physics explanations of the CDF 2022 measurement and the 2021 combined *a*_*μ*_ world average that originate from the same feature of the model, namely the combination of the *S*_1_ and *S*_3_ states through a non-vanishing mixing parameter, *λ*. For simplicity, we assume that only the couplings to muons that give the large chirality flipping enhancement from muon *g* − 2 i.e., ${y}_{R}^{t\mu }$ and ${y}_{L}^{b\mu }$ are non-vanishing in the new Yukawa coupling.

After EW symmetry breaking, we have four scalar LQs, one with an electromagnetic charge *Q* = 4/3, one with *Q* = − 2/3 and two with *Q* = 1/3. The *Q* = 1/3 states mix through the *λ* interaction resulting in mass eigenstates ${S}_{+}^{\pm 1/3}$ and ${S}_{-}^{\pm 1/3}$ with masses ${m}_{{S}_{+}}$ and ${m}_{{S}_{-}}$:

8 $$\left(\begin{array}{l}{S}_{1}^{\pm 1/3}\\ {S}_{3}^{\pm 1/3}\end{array}\right)=\left(\begin{array}{ll}{c}_{\phi }&{s}_{\phi }\\ -{s}_{\phi }&{c}_{\phi }\end{array}\right)\left(\begin{array}{l}{S}_{+}^{\pm 1/3}\\ {S}_{-}^{\pm 1/3}\end{array}\right).$$

where *ϕ* is the mixing angle. The masses ${m}_{{S}_{3}}$, ${m}_{{S}_{1}}$ and the mixing parameter *λ* can be obtained from ${m}_{{S}_{+}}$, ${m}_{{S}_{-}}$ and *ϕ* from

9 $$\delta=\frac{\lambda {v}^{2}}{2}={s}_{\phi }{c}_{\phi }({m}_{{S}_{-}}^{2}-{m}_{{S}_{+}}^{2})$$

10 $${m}_{{S}_{1}}^{2}={m}_{{S}_{+}}^{2}{c}_{\phi }^{2}+{m}_{{S}_{-}}^{2}{s}_{\phi }^{2}$$

11 $${m}_{{S}_{3}}^{2}={m}_{{S}_{+}}^{2}{s}_{\phi }^{2}+{m}_{{S}_{-}}^{2}{c}_{\phi }^{2}$$

where *v* = 246 GeV is the vacuum expectation value. We also define $\Delta m\equiv {m}_{{S}_{+}}-{m}_{{S}_{-}}$ as the mass splitting between the two mass eigenstates *S*_+_ and *S*_−_. This mass splitting generates a non-vanishing oblique correction to the *T* parameter at one-loop^90^,

12 $$T=\frac{3}{4\pi {s}_{W}^{2}}\frac{1}{{M}_{W}^{2}}\left[F\left({m}_{{S}_{3}},{m}_{{S}_{-}}\right){\cos }^{2}\phi+F\left({m}_{{S}_{3}},{m}_{{S}_{+}}\right){\sin }^{2}\phi \right]$$

with

13 $$F\left({m}_{1},{m}_{2}\right)={m}_{1}^{2}+{m}_{2}^{2}-\frac{2{m}_{1}^{2}{m}_{2}^{2}}{{m}_{1}^{2}-{m}_{2}^{2}}\log \left(\frac{{m}_{1}^{2}}{{m}_{2}^{2}}\right).$$

This function vanishes when the masses are degenerate, that is, $\mathop{\lim }_{{m}_{1}\to {m}_{2}}F({m}_{1},{m}_{2})=0$. When Δ*m* = 0, the custodial symmetry is restored, and the corrections to the *T* parameter vanish as ${m}_{{S}_{3}}={m}_{+}={m}_{-}$. The shift in *M*_*W*_ from the SM prediction can be related to the oblique *T* parameter via,

14 $$\Delta {M}_{W}^{2}\equiv {\left.{M}_{W}^{2}\right|}_{{{{{{{{\rm{BSM}}}}}}}}}-{\left.{M}_{W}^{2}\right|}_{{{{{{{{\rm{SM}}}}}}}}}=\frac{\alpha {c}_{W}^{4}{M}_{Z}^{2}}{{c}_{W}^{2}-{s}_{W}^{2}}T$$

where *c*_*W*_ and *s*_*W*_ are the cosine and sine of the Weinberg angle. There are, furthermore, contributions from *S* and *U* that are subdominant in our LQ model. We determine the *T* that is required to explain the CDF 2022 measurement from our EW global fits and use that in combination with eq. (12) to test if LQ scenarios can explain this data. We checked analytically and numerically that our calculation obeys decoupling, with the additional BSM contributions approaching zero in the limit of large LQ masses. We cross-checked eq. (12) with a full one-loop calculation of the *T* parameter using SARAH 4.14.3^91^, FeynArts 3.11^92^, FormCalc 9.9^93^ and LoopTools 2.16^94^, finding good agreement with the results using just eq. (12). With the same setup we also verified that the combined contributions from *S* and *U* to *M*_*W*_ are small and do not impact significantly on our results. Finally we also implemented this model in FlexibleSUSY^95–98^ using the same SARAH model file and the recently updated *M*_*W*_ calculation^98^ and again found reasonable agreement with the results of our analysis described above.

Whilst the mass splitting impacts the *W* mass, the mixing impacts muon *g* − 2. Indeed, the mixing between interaction eigenstates allows the physical mass eigenstates to have both left- and right-handed couplings to muons and induces chirality flipping enhancements to muon *g* − 2^90^

15 $$\delta {a}_{\mu }=-\frac{3{m}_{\mu }^{2}}{16{\pi }^{2}}\frac{{m}_{t}}{{m}_{\mu }}\sin 2\phi \,{y}_{L}{y}_{R}\left(\frac{G({x}_{t}^{+})}{{m}_{{S}^{+}}^{2}}-\frac{G({x}_{t}^{-})}{{m}_{{S}^{-}}^{2}}\right)$$

where ${x}_{t}^{\pm }={m}_{t}^{2}/{m}_{{S}_{\pm }}^{2}$, the loop function is *G*(*x*) = 1/3*g*_*S*_(*x*) − *g*_*F*_(*x*) with

16 $$\begin{array}{rcl}{g}_{S}(x)&=&\frac{1}{x-1}-\frac{\log x}{{(x-1)}^{2}}\\ {g}_{F}(x)&=&\frac{x-3}{2{(x-1)}^{2}}+\frac{\log x}{{(x-1)}^{3}},\end{array}$$

and we simplify our notation by letting ${y}_{L}\equiv {y}_{L}^{b\mu }$ and ${y}_{R}\equiv {y}_{R}^{t\mu }$. Note that in this case there is a cancellation between the contribution of the lighter and heavier mass eigenstates, which reduces the effect of the very large chirality flipping enhancement *m*_*t*_/*m*_*μ*_ somewhat. If we consider couplings between *S*_1_ state and left-handed muons as well, the contributions would be considerably enhanced, so this would simply make it easier to explain *a*_*μ*_ while having little or no impact on the *W* mass prediction.

**Fig. 3:**
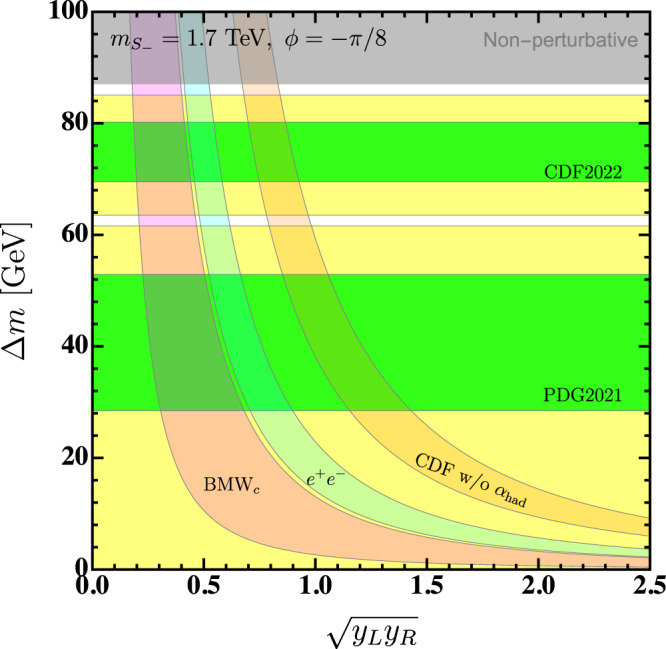
Scalar leptoquark explanation. Regions in the Δ*m* $-\sqrt{{y}_{L}{y}_{R}}$ plane of our LQ model that predict the *W*-boson mass and muon *g* − 2 in agreement with measurements. The mixing angle *ϕ* is set to be − *π*/8 and ${m}_{{S}_{-}}=1.7\,{{{{{{{\rm{TeV}}}}}}}}$. The grey region is excluded by the perturbativity requirement $|\lambda|\, < \, \sqrt{4\pi }$.

BSM contributions to *a*_*μ*_ and the *W* mass both require non-vanishing Δ*m*. For *a*_*μ*_, it further requires non-vanishing mixing *ϕ* and relies on *y*_*L*_*y*_*R*_. Thus it is possible to find explanations of both *a*_*μ*_ and the 2022 CDF measurement of *M*_*W*_ by varying *y*_*L*_*y*_*R*_ and Δ*m* with non-zero mixing angle *ϕ*. In Fig. 3 we show regions in the Δ*m*–$\sqrt{{y}_{L}{y}_{R}}$ plane that explain both measurements, where we have fixed the LQ mass to 1.7 TeV, a little above the LHC limit, and we also fixed the mixing angle *ϕ* = − *π*/8.

LQ couplings of greater than about a half can explain the *a*_*μ*_measurement within 1*σ* when we use the SM prediction from the theory white paper, where *e*^+^*e*^−^ data is used for ${a}_{\mu }^{{{{{{{{\rm{HVP}}}}}}}}}$. Explaining the SM prediction from the BMW collaboration requires even smaller couplings, though in this case the tension with the SM is anyway <2*σ*. Using *e*^+^*e*^−^ data to also fix Δ*α*_had_ means there then remains an additional deviation between the SM *M*_*W*_ prediction and the measured values. To explain the new 2022 CDF result with BSM contributions as well, Δ*m* ≈ 75 GeV is then required, and the dual explanation of the *M*_*W*_ and *a*_*μ*_ anomalies can be achieved in the region where the green CDF 2022 band overlaps with the light blue *e*^+^*e*^−^ band in Fig. 3. The deviation between the SM prediction for the *W* boson mass and the 2021 PDG value is not so large and within 2*σ* it does not need new physics contributions, so the yellow 2*σ* band for this in Fig. 3 can extend to Δ*m* ≈ 0, but to within 1*σ* a small non-zero Δ*m* is required. Further, the interaction coupling *λ* is proportional to the mass splitting Δ*m* with fixed mixing angle *ϕ*. In order to keep the coupling perturbative ($|\lambda|\, < \, \sqrt{4\pi }$), there is an upper limit on the mass splitting as shown by the grey band in Fig. 3. Note that the region that can accommodate the CDF measurement is close to the non-perturbative region, as the CDF measurement requires large mass splitting. However, it is still possible to explain the new *M*_*W*_ measurement within 1*σ* in the perturbative region.

### Further constraints

This model establishes a proof of principle of a simple, dual explanation of both anomalies. There remains, however, the question of whether this model or extensions of it can simultaneously explain recent flavour physics measurements and anomalies and satisfy additional phenomenological constraints. The latter may be particularly severe as the required Yukawa couplings are ${{{{{{{\mathcal{O}}}}}}}}(1)$.

For example, the recently measured branching ratio BR(*h* → *μμ*)^99,100^ can be an important probe of leptoquark explanations of muon *g* − 2^101,102^. Reference ^101^ showed that when you have *S*_1_ and *S*_3_ with only right-handed couplings for *S*_1_ there is already a significant tension with the current measurements. There are several ways to avoid this tension. If the leptoquarks are embedded in a more fundamental theory there could be additional light states that result in cancellations with the leptoquark contribution to *h* → *μμ*, for example through destructive interference between tree- and loop-level diagrams. This can be achieved by extending the LQ model in the framework of the two-Higgs-doublet model in the wrong-sign Yukawa coupling region^103^. Alternatively we can reintroduce the left-handed coupling of the *S*_1_ state, which brings two benefits.

First, allowing significant left-handed couplings from *S*_1_ substantially reduces the size of the Yukawa couplings needed to explain muon *g* − 2 (as stated earlier). We show in the Supplementary Note 3 that, as can be anticipated from ref. ^101^, this makes it possible to satisfy the BR(*h* → *μμ*) data while simultaneously explaining muon *g* − 2, while keeping the mass splitting fixed to a value required to explain the CDF measurement of the *W* mass. Second introducing this coupling gives additional freedom that can help explain the well-known anomalies of Lepton Flavour Universality Violation, while avoiding other limits from flavour physics.

Indeed, the severe constraints from *μ* → *eγ*, *a*_*e*_, etc. can all be evaded by allowing the proper flavour ansatz for the Yukawa couplings^104^. At the same time the ${R}_{{K}^{*}}$ and ${R}_{{D}^{*}}$ anomalies can be explained via extra tree-level processes from the scalar LQ with *Q* = 4/3 to *b* → *sμ*^+^*μ*^−^ and two scalar LQs with *Q* = 1/3 to *b* → *cτν*. Although the latter two scalar LQs also contribute to ${R}_{{K}^{*}}^{\nu \nu }$ through tree-level process *b* → *sνν*, these enhancements are under control and their effects are not in conflict with the current measurement^104^.

Finally, we show that an explanation of the *W* mass in our model must be accompanied by new physics below about 2 TeV. In order to explain the CDF measurement at 2*σ* with *S* = 0 (which is a good approximation in our model), we need 0.14 ≲ *T* ≲ 0.17. Expanding eq. (12),

17 $$T\simeq \frac{{\lambda }^{2}{v}^{4}}{16\pi {m}_{-}^{2}{M}_{W}^{2}{s}_{W}^{2}}$$

such that combining the lower limit on the *T* parameter and the perturbativity limit $\lambda \le \sqrt{4\pi }$ we obtain

18 $${m}_{-}\, \lesssim\, 2\,{{{{{{{\rm{TeV}}}}}}}}$$

As the mass splitting, Δ*m* = *m*_+_ − *m*_−_ ≲ 100 GeV, our model thus predicts two *Q* = 1/3 LQ states below about 2 TeV.

## Supplementary information

Supplementary Information

## Data Availability

The datasets generated during and/or analysed during the current study are available from the corresponding author on request.

## Code availability

The custom computer codes used to generate results are available from the corresponding author on request.
