# Supplementary material for: Hadronic uncertainties versus new physics for the W boson mass and Muon g − 2 anomalies
Source: Nat Commun. 2023 Feb 7;14:659. doi: 10.1038/s41467-023-36366-7 (PMC9902446; doi:10.1038/s41467-023-36366-7)
Supplement: Supplementary file 1 — Supplementary Information [file 41467_2023_36366_MOESM1_ESM.pdf]

Supplementary Information
Hadronic Uncertainties versus New Physics for the W boson Mass and Muon $g-2$ Anomalies

Peter Athron[1 *], Andrew Fowlie[1 †], Chih-Ting Lu[1 ‡], Lei Wu[1 §], Yongcheng Wu[1 ¶], Bin Zhu[2 **]

[1]Department of Physics and Institute of Theoretical Physics, Nanjing Normal University, Nanjing, 210023, China

[2]Department of Physics, Yantai University, Yantai 264005, China

[*]peter.athron@njnu.edu.cn [†]andrew.j.fowlie@njnu.edu.cn [‡]06285@njnu.edu.cn

[§]leiwu@njnu.edu.cn [¶]ycwu@njnu.edu.cn [**]zhubin@mail.nankai.edu.cn

In this supplemental material, we present the data in our global fits (Supplementary Table 1), our procedure of our simple combination of M_W and the transformation between $\Delta\alpha_{\rm had}$ and $a_\mu^{\rm HVP}$.

Supplementary Note 1 - Simple combination of W mass measurements

We compute a weighted average of N measurements following a standard procedure (see e.g., Ref. [1]),

$$\bar{x} \pm \Delta x = \frac{\sum_{i=1}^{N} w_i x_i}{\sum_{i=1}^{N} w_i} \pm \left(\sum_{i=1}^{N} w_i\right)^{-1/2} \tag{1}$$

where $w_i = 1/\sigma_i^2$. We include the seven measurements — LEP [2], LHCb [3], ATLAS [4], D0 [5] 92-95 (95/pb) and 02-09 (4.3/fb), CDF [6, 7, 8] 88-95 (107/pb) and 02-11 (9.1/fb) — avoiding double-counting the CDF data. This resulted in our simple combination

$$M_W = 80.411 \pm 0.007\,\text{GeV}. \tag{2}$$

This combination remains about 6σ away from the SM. The chi-squared associated with this estimate,

$$\chi^2 = \sum_{i=1}^{N} w_i (x_i - \bar{x})^2, \tag{3}$$

was 17.7 with $N-1=6$ degrees of freedom. The associated significance was 2.5σ, found from

$$p = 1 - F_{\chi^2_6}(\chi^2) \quad \text{and} \quad Z = \Phi^{-1}(1-p) \tag{4}$$

where $F_{\chi^2_n}$ is the chi-squared cumulative density function with n degrees of freedom and Φ is the standard normal cumulative density function. This result depends on the number and

choices of measurements combined. The code to reproduce these calculations is available at .

We note, however, that a true combination should include careful consideration of correlated systematic errors and expert judgment about unstated or underestimated errors and systematics. For example, if the reduced chi-squared indicates discrepant measurements, the PDG [1] may inflate the estimated errors, though this does not impact the central value, or decline to combine them (for further discussion see e.g., Ref. [9, 10, 11, 12]). In our case, the PDG prescription would inflate the error by about two, if the measurements were combined. This would reduce the discrepancy with the SM to about 3σ.

Supplementary Note 2 - The relationship between $\Delta\alpha_{\text{had}}$ and a_μ^{HVP}

Since both $\Delta\alpha_{\text{had}}$ and a_μ^{HVP} can be extracted from σ_{had} measurements, changes in σ_{had} affect the transformation between $\Delta\alpha_{\text{had}}$ and a_μ^{HVP}. On the one hand, one can directly use the experimental data from σ_{had} measurements (the e^+e^- data) to derive $\Delta\alpha_{\text{had}}$ and a_μ^{HVP} with the data-driven method as shown in Equation (2) and Equation (3) in the main text. On the other hand, we can extract $\Delta\alpha_{\text{had}}$ from EW fits or from estimates of the HVP contributions from the BMW lattice calculation and use that to indirectly indicate possible changes in σ_{had} compared with the experimental measurements. For the latter, we must make assumptions about the energy dependence of σ_{had} and the energy range in which it changes.

As pointed out in Ref. [13], we could modify σ_{had} only in the energy ranges:

$$m_{\pi_0} \leq \sqrt{s} \leq 1.937\,\text{GeV}, \tag{5}$$
$$m_{\pi_0} \leq \sqrt{s} \leq 11.199\,\text{GeV or} \tag{6}$$
$$m_{\pi_0} \leq \sqrt{s} \leq \infty, \tag{7}$$

that is, at low energies, at any moderate energies, or across the entire energy range. The hadronic cross section is unchanged above these thresholds. We previously only considered the latter possibility Supplementary Equation (7); we now consider Supplementary Equation (5), Supplementary Equation (6) and reconsider the relationship between $\Delta\alpha_{\text{had}}$ and a_μ^{HVP}, i.e. we follow the procedure introduced in Ref. [13]. We find that using Supplementary Equation (7) and transforming $\Delta\alpha_{\text{had}}$ to a_μ^{HVP} (a_μ^{HVP} to $\Delta\alpha_{\text{had}}$) results in the most conservative (aggressive) deviation from the e^+e^- data. However, the low energy range projection Supplementary Equation (5) shows the opposite behaviour. We assume that σ_{had} changes by an overall factor over the range m_{π_0} to infinity, Supplementary Equation (7). This is scenario (3) in Ref. [13]. Based on this assumption, we derive a_μ^{HVP} from $\Delta\alpha_{\text{had}}$ (and vice-versa) with a naive and uniform scaling of the cross-section from the e^+e^- data [14, 15]. In this way, we obtain alternative predictions for a_μ^{HVP} that correspond to the M_W measurement under the assumption that no new physics affects the EW fits.

We use the above method to alter the SM prediction for a_μ by taking all contributions except for a_μ^{HVP} to be those used in Ref. [14]. We then determine the deviation between the combined 2021 world average and our predictions (δa_μ), after combining both theoretical and experimental uncertainties. The experimental uncertainty is fixed to 41×10^{-11} [16], but the theoretical uncertainty depends on the way in which a_μ^{HVP} was chosen. Each δa_μ is indicated on the right-hand side of Figure 2 in the main text and listed in Table I in the

main text where we also show how many standard deviations this represents. Finally, we visualize δa_μ and its tension from Table I in the main text in Supplementary Figure **1**.

Lastly, to complete our study of the transformation between $\Delta\alpha_{\rm had}$ and $a_\mu^{\rm HVP}$, we consider case Supplementary Equation (5). Since the BMW collaboration only released the first two bins data ($0\,\text{GeV} < \sqrt{s} \leq 1\,\text{GeV}$ and $1\,\text{GeV} < \sqrt{s} \leq \sqrt{10}\,\text{GeV}$) of $\Delta\alpha_{\rm had}$ [17], case Supplementary Equation (5) may be suggested by BMWc results. Therefore, we include this case in Supplementary Table 2 for readers as a reference. Note we only use the integral breakdown from Ref. [15, 18] which provided smaller uncertainties for $a_\mu^{\rm HVP}$ from the e^+e^- data in their calculations. Hence, the δa_μ in Supplementary Table 2 would be reduced if the integral breakdown from other references [19, 20] was used. A significant caveat to keep in mind is that we don't definitively know whether case Supplementary Equation (5), Supplementary Equation (6) or Supplementary Equation (7) should be preferred.

Supplementary Note 3 - The correlation between BR($h \to \mu\mu$) and muon $(g-2)$

As discussed in Further constraints in the main text, measurements of the branching ratio BR($h \to \mu\mu$) [21, 22] severely constrain our simplified leptoquark model. Relaxing our simplifications by reintroducing left-handed couplings of the S_1 state, λ_L, substantially reduces the size of the Yukawa couplings needed to explain muon $g-2$. In Supplementary Figure **2** we show that this alleviates tension between $g-2$ and BR($h \to \mu\mu$). The leptoquark model explains muon $g-2$ while satisfying experimental upper limits on BR($h \to \mu\mu$) for $\lambda_L \gtrsim 10^{-2}$. The branching ratio predictions may be SM-like for $\lambda_L \simeq 5 \times 10^{-2}$. Nevertheless, the model typically predicts deviations from the SM-predictions for BR($h \to \mu\mu$) that may be observable in the future.

Supplementary Tables

Parameter	Measured value	Ref.
PDG 2021 M_W [GeV]	80.379(12)	[23]
CDF 2022 M_W [GeV]	80.4335(94)	[8]
$\Delta\alpha^{(5)}_{\rm had}(M_Z^2)$	See text	
m_h [GeV]	125.25(17)	[23]
m_t [GeV][1]	172.76(58)	[23]
$\alpha_s(M_Z)$	0.1179(9)	[23]
Γ_W [GeV]	2.085(42)	[23]
Γ_Z [GeV]	2.4952(23)	[24]
M_Z [GeV]	91.1875(21)	[24]
$A^{0,b}_{\rm FB}$	0.0992(16)	[24]
$A^{0,c}_{\rm FB}$	0.0707(35)	[24]
$A^{0,\ell}_{\rm FB}$	0.0171	[24]
A_b	0.923(20)	[24]
A_c	0.670(27)	[24]
A_ℓ(SLD)	0.1513(21)	[24]
A_ℓ(LEP)	0.1465(33)	[24]
R^0_b	0.21629(66)	[24]
R^0_c	0.1721(30)	[24]
R^0_ℓ	20.767(25)	[24]
σ^0_h [nb]	41.540(37)	[24]
$\sin^2\theta^\ell_{\rm eff}(Q_{\rm FB})$	0.2324(12)	[24]
$\sin^2\theta^\ell_{\rm eff}$(Teva)	0.23148(33)	[25]
$\overline{m}_c$ [GeV]	1.27(2)	[23]
$\overline{m}_b$ [GeV]	$4.18^{(3)}_{(2)}$	[23]

Supplementary Table 1: The measurements included in the global EW fit. Correlations among $(M_Z, \Gamma_Z, \sigma^0_h, R^0_\ell, A^{0,\ell}_{\rm FB})$ and among $(A^{0,c}_{\rm FB}, A^{0,b}_{\rm FB}, A_c, A_b, R^0_c, R^0_b)$ are also taken into account [24].

	M_W	Indirect			PDG 2021			CDF 2022			Simple Combination		
	$\Delta\alpha_{\rm had}$	BMWc	e^+e^-	Indirect	BMWc	e^+e^-	Indirect	BMWc	e^+e^-	Indirect	BMWc	e^+e^-	Indirect
Input	M_W [GeV]	-	-	-	80.379(12)	80.379(12)	80.379(12)	80.4335(94)	80.4335(94)	80.4335(94)	80.411(7)	80.411(7)	80.411(7)
	$\Delta\alpha^{(5)}_{\rm had}(M_Z^2) \times 10^4$	277.4(1.2)	276.1(1.1)	-	277.4(1.2)	276.1(1.1)	-	277.4(1.2)	276.1(1.1)	-	277.4(1.2)	276.1(1.1)	-
Fitted	χ^2/dof	16.28/15	16.01/15	15.89/14	19.51/16	18.74/16	17.59/15	65.07/16	62.58/16	47.19/15	52.34/16	49.79/16	35.48/15
	M_W [GeV]	80.355(6)	80.357(6)	80.359(9)	80.360(6)	80.361(6)	80.367(7)	80.379(5)	80.380(5)	80.396(7)	80.380(5)	80.381(5)	80.393(6)
	$\Delta\alpha_{\rm had} \times 10^4$	277.1(1.2)	275.9(1.1)	274.4(4.4)	276.8(1.1)	275.6(1.1)	271.7(3.8)	275.6(1.1)	274.7(1.0)	260.9(3.6)	275.6(1.1)	274.6(1.0)	262.3(3.4)
	$\delta a_\mu \times 10^{11}$	-	-	438(396)	173(54)	306(54)	748(339)	306(54)	416(54)	1997(320)	306(54)	416(54)	1776(301)
	Tension	-	-	1.1σ	3.2σ	5.7σ	2.2σ	5.7σ	7.7σ	6.2σ	5.7σ	7.7σ	5.9σ
	δM_W [MeV]	79(11)	77(11)	75(13)	74(11)	73(11)	67(12)	55(11)	54(11)	38(12)	54(11)	53(11)	41(11)
	Tension	7.2σ	7.0σ	5.8σ	6.7σ	6.6σ	5.6σ	5.0σ	4.9σ	3.2σ	4.9σ	4.8σ	3.7σ

Supplementary Table 2: SM predictions from EW fits for $\Delta\alpha_{\rm had}$ and M_W, and the differences with respect to measurements of muon $g-2$ and the W mass, δa_μ and $\delta M_W \equiv M_W^{\rm CDF} - M_W$ using the low energy projection Supplementary Equation (5) for the transformation between $\Delta\alpha_{\rm had}$ and $a_\mu^{\rm HVP}$.

Supplementary Figures

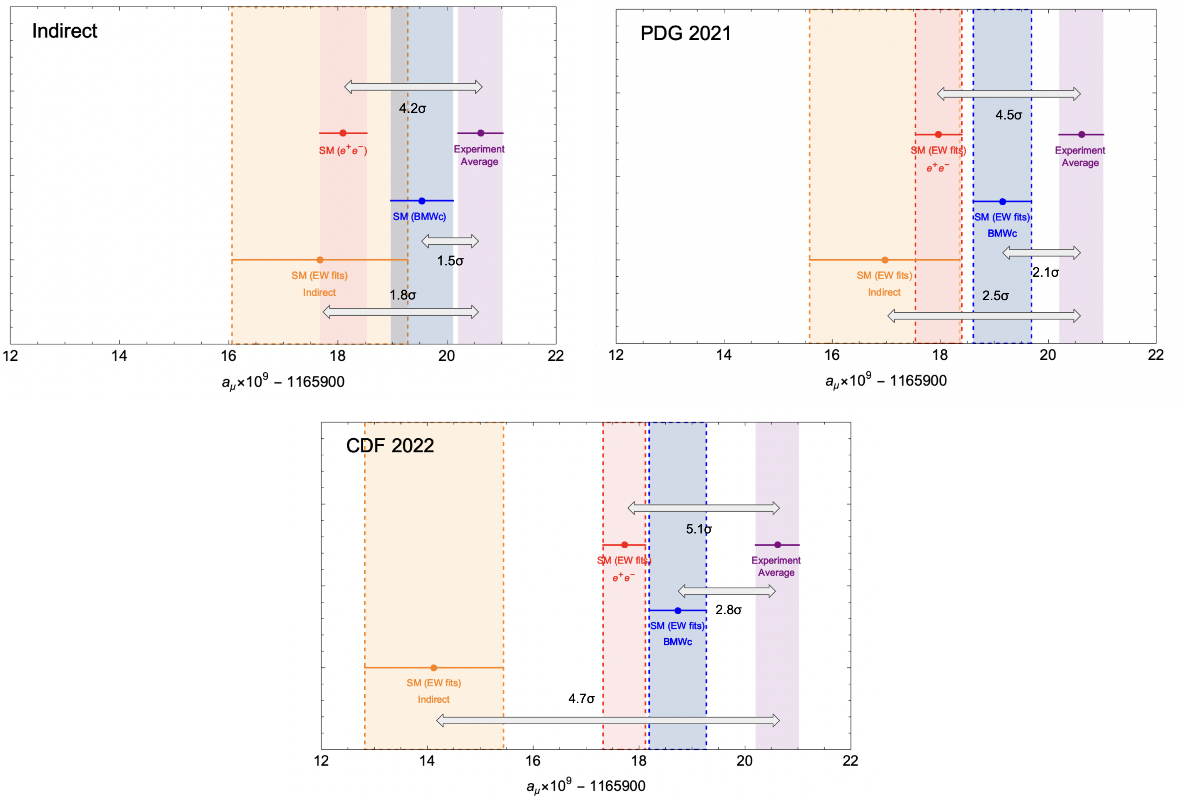

Supplementary Figure 1: **The a_μ in different fitting scenarios and corresponding tensions with experiment average**. The a_μ from experiment average of FNAL E989 and BNL E821 (purple), SM predictions from BMWc (blue), e^+e^- data (red), EW fits w/o $\Delta\alpha_{\rm had}$ (orange-dashed), EW fits with $\Delta\alpha_{\rm had}$ from e^+e^- (red-dashed), EW fits with $\Delta\alpha_{\rm had}$ from BMWc (blue-dashed). The tensions of δa_μ are also shown for the comparison. The upper-left panel is w/o M_W input, the upper-right and bottom panels are with PDG 2021 and CDF 2022 M_W inputs, respectively.

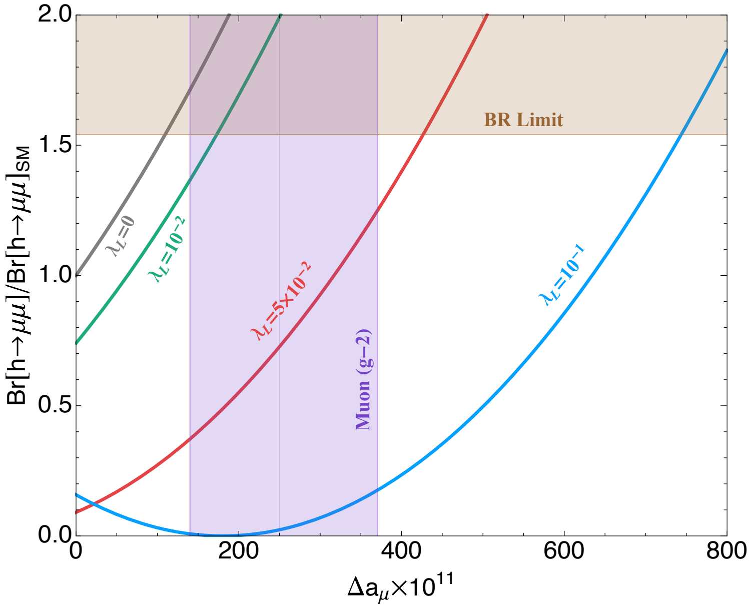

Supplementary Figure 2: Correlation between the muon $g-2$ and BR$(h \to \mu\mu)$. Correlation between the muon $g-2$ and the branching ratio BR$(h \to \mu\mu)$ normalized to its SM value in our leptoquark model. The gray line shows the relationship without the contribution from left-handed couplings ($\lambda_L = 0$), while the green, red, and blue lines correspond to $\lambda_L = 10^{-2}$, 5×10^{-2} and 10^{-1}, respectively. The mass splitting is fixed at 30 GeV to be compatible with the W mass measurement. The upper limit on the branching ratio excludes the brown region, whereas the required muon $g-2$ is shown by the purple region.

References

[1] Zyla, P. *et al.* Review of Particle Physics. *PTEP* **2020**, 083C01 (2020).

[2] Schael, S. *et al.* Electroweak Measurements in Electron-Positron Collisions at W-Boson-Pair Energies at LEP. *Phys. Rept.* **532**, 119–244 (2013). 1302.3415.

[3] Aaij, R. *et al.* Measurement of the W boson mass. *JHEP* **01**, 036 (2022). 2109.01113.

[4] Aaboud, M. *et al.* Measurement of the W-boson mass in pp collisions at $\sqrt{s} = 7$ TeV with the ATLAS detector. *Eur. Phys. J. C* **78**, 110 (2018). [Erratum: Eur. Phys. J. C **78**, 898 (2018)], 1701.07240.

[5] Abazov, V. M. *et al.* Measurement of the W Boson Mass with the D0 Detector. *Phys. Rev. Lett.* **108**, 151804 (2012). 1203.0293.

[6] Aaltonen, T. *et al.* Precise measurement of the W-boson mass with the CDF II detector. *Phys. Rev. Lett.* **108**, 151803 (2012). 1203.0275.

[7] Aaltonen, T. A. *et al.* Combination of CDF and D0 W-Boson Mass Measurements. *Phys. Rev. D* **88**, 052018 (2013). 1307.7627.

[8] Aaltonen, T. *et al.* High-precision measurement of the W boson mass with the CDF II detector. *Science* **376**, 170–176 (2022).

[9] Youden, W. J. Enduring values. *Technometrics* **14**, 1–11 (1972).

[10] Taylor, B. N. Numerical Comparisons of Several Algorithms for Treating Inconsistent Data in a Least-Squares Adjustment of the Fundamental Constants (1982). URL https://nvlpubs.nist.gov/nistpubs/Legacy/IR/nbsir81-2426.pdf.

[11] Barlow, R. Systematic errors: Facts and fictions. In *Conference on Advanced Statistical Techniques in Particle Physics*, 134–144 (2002). hep-ex/0207026.

[12] Jeng, M. Bandwagon effects and error bars in particle physics. *Nucl. Instrum. Meth. A* **571**, 704–708 (2007).

[13] Crivellin, A., Hoferichter, M., Manzari, C. A. & Montull, M. Hadronic Vacuum Polarization: $(g-2)_\mu$ versus Global Electroweak Fits. *Phys. Rev. Lett.* **125**, 091801 (2020). 2003.04886.

[14] Aoyama, T. *et al.* The anomalous magnetic moment of the muon in the Standard Model. *Phys. Rept.* **887**, 1–166 (2020). 2006.04822.

[15] Keshavarzi, A., Nomura, D. & Teubner, T. Muon $g-2$ and $\alpha(M_Z^2)$: a new data-based analysis. *Phys. Rev. D* **97**, 114025 (2018). 1802.02995.

[16] Abi, B. *et al.* Measurement of the Positive Muon Anomalous Magnetic Moment to 0.46 ppm. *Phys. Rev. Lett.* **126**, 141801 (2021). 2104.03281.

[17] Borsanyi, S. *et al.* Leading hadronic contribution to the muon magnetic moment from lattice QCD. *Nature* **593**, 51–55 (2021). 2002.12347.

[18] Keshavarzi, A., Nomura, D. & Teubner, T. $g-2$ of charged leptons, $\alpha(M_Z^2)$, and the hyperfine splitting of muonium. *Phys. Rev. D* **101**, 014029 (2020). 1911.00367.

[19] Davier, M., Hoecker, A., Malaescu, B. & Zhang, Z. Reevaluation of the hadronic vacuum polarisation contributions to the Standard Model predictions of the muon $g-2$ and $\alpha(m_Z^2)$ using newest hadronic cross-section data. *Eur. Phys. J. C* **77**, 827 (2017). 1706.09436.

[20] Davier, M., Hoecker, A., Malaescu, B. & Zhang, Z. A new evaluation of the hadronic vacuum polarisation contributions to the muon anomalous magnetic moment and to $\boldsymbol{\alpha}(\mathbf{m_Z^2})$. *Eur. Phys. J. C* **80**, 241 (2020). [Erratum: Eur. Phys. J. C **80**, 410 (2020)], 1908.00921.

[21] Aad, G. *et al.* A search for the dimuon decay of the Standard Model Higgs boson with the ATLAS detector. *Phys. Lett. B* **812**, 135980 (2021). 2007.07830.

[22] Sirunyan, A. M. *et al.* Evidence for Higgs boson decay to a pair of muons. *JHEP* **01**, 148 (2021). 2009.04363.

[23] Zyla, P. A. *et al.* Review of Particle Physics. *PTEP* **2020**, 083C01 (2020).

[24] Schael, S. *et al.* Precision electroweak measurements on the Z resonance. *Phys. Rept.* **427**, 257–454 (2006). hep-ex/0509008.

[25] Aaltonen, T. A. *et al.* Tevatron Run II combination of the effective leptonic electroweak mixing angle. *Phys. Rev. D* **97**, 112007 (2018). 1801.06283.
